# Mechanistic Study on Dual-Donor Modified Distyryl-BODIPY Photosensitizers

**DOI:** 10.3390/ma19173700

**Published:** 2026-08-31

**Authors:** Yunlong Yang, Di Wang, Xue Ma, Jiaye Yang, Guanghui Cheng, Jianfang Cao

**Affiliations:** 1 School of Chemical Engineering, Ocean and Life Sciences, Panjin Campus, Dalian University of Technology, Panjin 124221, China; dragonclouds@mail.dlut.edu.cn (Y.Y.); wangdi0072@mail.dlut.edu.cn (D.W.); 13941074037@163.com (X.M.); 2 Leicester International Institute, Panjin Campus, Dalian University of Technology, Panjin 124221, China; yangjiaye11@icloud.com; 3 Department of Energy Chemistry and Materials Engineering, Shanxi Institute of Energy, Jinzhong 030600, China; chenggh@sxie.edu.cn

**Keywords:** distyryl-BODIPY, photosensitizers, intersystem crossing, charge transfer, photodynamic therapy (PDT), heavy-atom-free

## Abstract

This work systematically investigates how different electron-donating groups modulate the photophysical properties and singlet-oxygen quantum yields of donor–acceptor (D–A) distyryl boron-dipyrromethene (BODIPY) photosensitizers, with the donors attached at the para positions of both styryl arms. The study focuses on three compounds that have been previously reported in the literature (M1 with a dimethylacridine donor, M2 with a phenoxazine donor, and M3 with a phenothiazine donor), yet only experimental characterizations are available for them. Using computational chemistry methods, we comprehensively analyzed their geometric configurations, spectral features, spin–orbit coupling (SOC) effects, and electron–hole orbital distributions. The calculated results reveal that M2 possesses the lowest oxidation potential and thus the strongest electron-donating ability among the three donors. Compared with M1 and M3, M2 exhibits larger SOC values, a smaller energy gap between the excited singlet and triplet states, and the highest intersystem crossing (ISC) rate constant. Notably, the energy gaps ΔE (T_1_ → S_0_) for all three molecules exceed 0.98 eV, which is thermodynamically favorable for sensitizing triplet oxygen to singlet oxygen. Moreover, each of M1–M3 can form dual charge-transfer singlet states, affording two distinct singlet charge-transfer (^1^CT) → triplet locally excited (^3^LE) ISC pathways. This theoretical work demonstrates that dual-donor modification can generate dual charge-transfer states and donor strength serves as an important regulating factor for the intersystem-crossing performance of the three investigated distyryl-BODIPY derivatives. The presence of such dual ISC channels offers a new strategic avenue for the rational design of highly efficient photosensitizers from a theoretical perspective, although further experimental validation in biological systems is required to confirm their therapeutic applicability.

## 1. Introduction

Conventional cancer therapies often suffer from severe side effects, limited efficacy against advanced solid tumors, and drug resistance induced by the tumor microenvironment, making them inadequate for treating metastatic lesions [1,2]. In this context, photodynamic therapy (PDT) has emerged as a promising alternative owing to its low toxicity and high targeting capability, and it has attracted growing interest in antitumor research [3,4,5,6]. PDT relies on the photochemical reaction among a photosensitizer, specific light, and oxygen to generate singlet oxygen for precise destruction of malignant cells, with minimal damage to normal tissues, and it can also disrupt tumor vasculature and activate antitumor immunity [7]. The photosensitizer is the core determinant of therapeutic efficacy; however, conventional photosensitizers often suffer from limited tissue penetration and cannot simultaneously achieve fluorescence imaging and reactive oxygen species (ROS) generation, hindering theranostic applications [8]. Upon photoexcitation, photosensitizers undergo two competing deactivation pathways: radiative decay (fluorescence) and intersystem crossing (ISC) to the triplet state for ROS production. Consequently, fluorescence quantum yield (Φ_f_) and singlet-oxygen quantum yield (Φ_Δ_) are intrinsically in a trade-off relationship [9]. Moreover, free intramolecular rotation in long-wavelength fluorescent dyes can dissipate excited-state energy, further reducing fluorescence efficiency [10].

The key challenge in developing high-performance photosensitizers lies in balancing Φ_f_ and Φ_Δ_. Quantum chemical calculations have systematically elucidated the structure–property relationships and electronic modulation strategies for tuning charge-transfer behavior and ISC efficiency through molecular design, conformation control, and donor–acceptor (D-A) engineering [11,12,13,14]. Previous studies have demonstrated that distyryl-BODIPY derivatives, obtained by introducing styryl conjugates at the 3,5-positions of the BODIPY core, exhibit excellent photophysical properties and abundant modification sites [15,16]. A series of distyryl-BODIPY derivatives bearing various arylamine donors at the terminal positions of the styryl groups have been synthesized and fully characterized [15]; other work on multipolar derivatives has further confirmed that side-chain modifications can flexibly tune absorption and emission properties [17]. However, BODIPY photosensitizers with only a single electron-donating group at the *meso* position often show limited ISC efficiency and cannot achieve high ROS generation [18]. Experimental data indicate that heavy-atom-free BODIPYs with a single arylamine donor at the *meso* position generally exhibit low Φ_Δ_ values [19], and theoretical calculations have further revealed that such monosubstituted molecules have weak charge-transfer character and only a single ISC channel, preventing efficient triplet accumulation [10].

Although computational studies have shown that various electron-donating groups can modulate mixed CT/LE excited states and improve triplet population, a systematic comparative analysis of how different donor structures govern the photophysical evolution in D-A distyryl-BODIPY photosensitizers is still lacking, and the intrinsic structure–activity relationships involving heteroatom-induced charge transfer and ISC remain to be clarified [20]. To address this gap, we selected three experimentally reported compounds, M1–M3, for a comprehensive mechanistic investigation [21]. By calculating the oxidation potentials of the donor moieties, we found that M2 exhibits the lowest oxidation potential and thus the strongest electron-donating ability. Electron density difference maps and hole–electron distribution analyses visually confirm that the ISC processes in all three molecules are governed by the ^1^CT → ^3^LE mechanism. Notably, the S_2_ and S_3_ excited states of M1 are quasi-degenerate, reducing the available ISC channels and resulting in lower ISC efficiency compared with M2 and M3. Frontier molecular orbital analysis reveals that M1 has the highest orbital overlap and oscillator strength, leading to the fastest radiative decay rate and the highest fluorescence quantum yield among the three.

## 2. Materials and Methods

To ensure reliable theoretical elucidation of the mechanisms underlying the dual functions of D–A BODIPY photosensitizers as imaging and therapeutic agents, we performed density functional theory (DFT) and time-dependent DFT (TD-DFT) calculations using the Gaussian 16 package [22]. Geometry optimizations and frequency analyses were carried out with the M06-2X functional [23] and the 6-31G(d,p) basis set [24,25], and solvation effects were modeled by the SMD implicit solvent model [26]. Spin–orbit coupling (SOC) values between singlet and triplet excited states were computed with the BDF program (DS-BDF_2022A) [27,28,29,30,31,32], while intersystem crossing (ISC) rate constants were obtained using the MOMAP software (version 2021B) [33,34,35,36,37,38,39]. CT and LE characters, natural transition orbitals (NTOs), and net charge transfer between donor and acceptor moieties were evaluated with the Multiwfn(version 3.8 ) program and visualized with VMD (6.5.0) [40]. In addition, oxidation potentials of donors and reduction potentials of acceptors were calculated using the ORCA (orca version 4.2.1) package [41,42,43,44]. This comprehensive computational protocol provides a solid foundation for understanding the electronic structures, photophysical properties, and excited-state interactions of these photosensitizer molecules, which is essential for revealing their dual-function potential.

The M06-2X functional was selected because it has been widely used and validated for studying excited-state properties, CT and LE states in organic donor–acceptor systems and BODIPY derivatives [23,45]. To further verify the reliability of this level of theory for the specific distyryl-BODIPY derivatives investigated in this work, we directly compared the calculated absorption/emission wavelengths, fluorescence quantum yields, and singlet-oxygen quantum yields for M1–M3 with the available experimental data (Table S1 in Supporting Information). The satisfactory agreement between the calculated and measured photophysical parameters demonstrates that the M06-2X/6-31G(d,p) scheme is suitable for the photophysical calculations of the present distyryl-BODIPY derivatives.

## 3. Results and Discussion

### 3.1. Geometrically Optimized Structures of M1–M3 at the S_0_, S_n_, and T_n_ States, Together with the Oxidation Potentials of Their Donor Moieties

**Figure 1:**
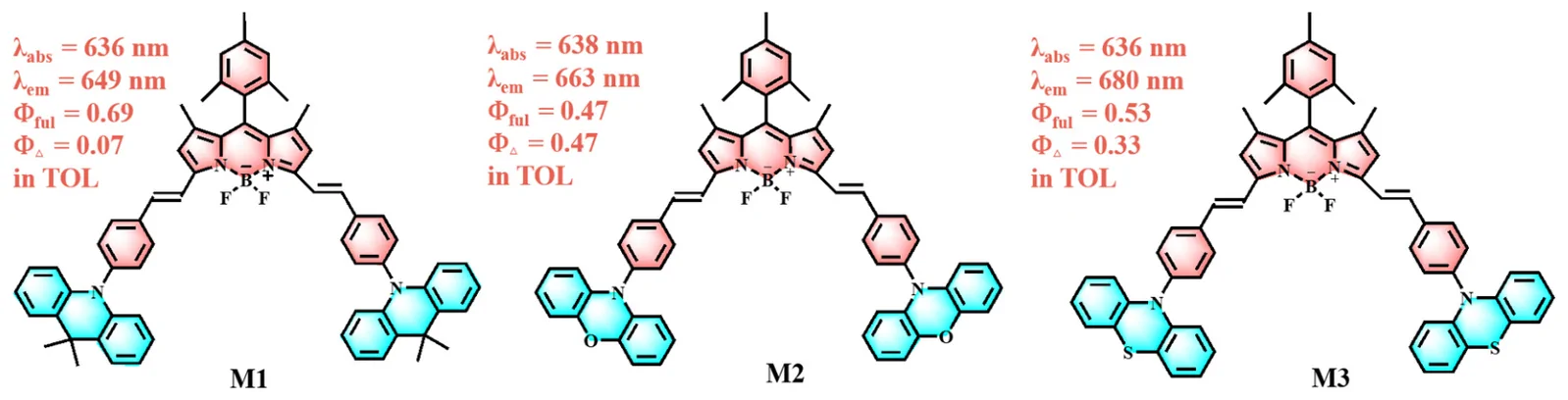
The chemical structures and photophysical properties of M1—M3 (acceptor in pink, donor in blue).

**Figure 2:**
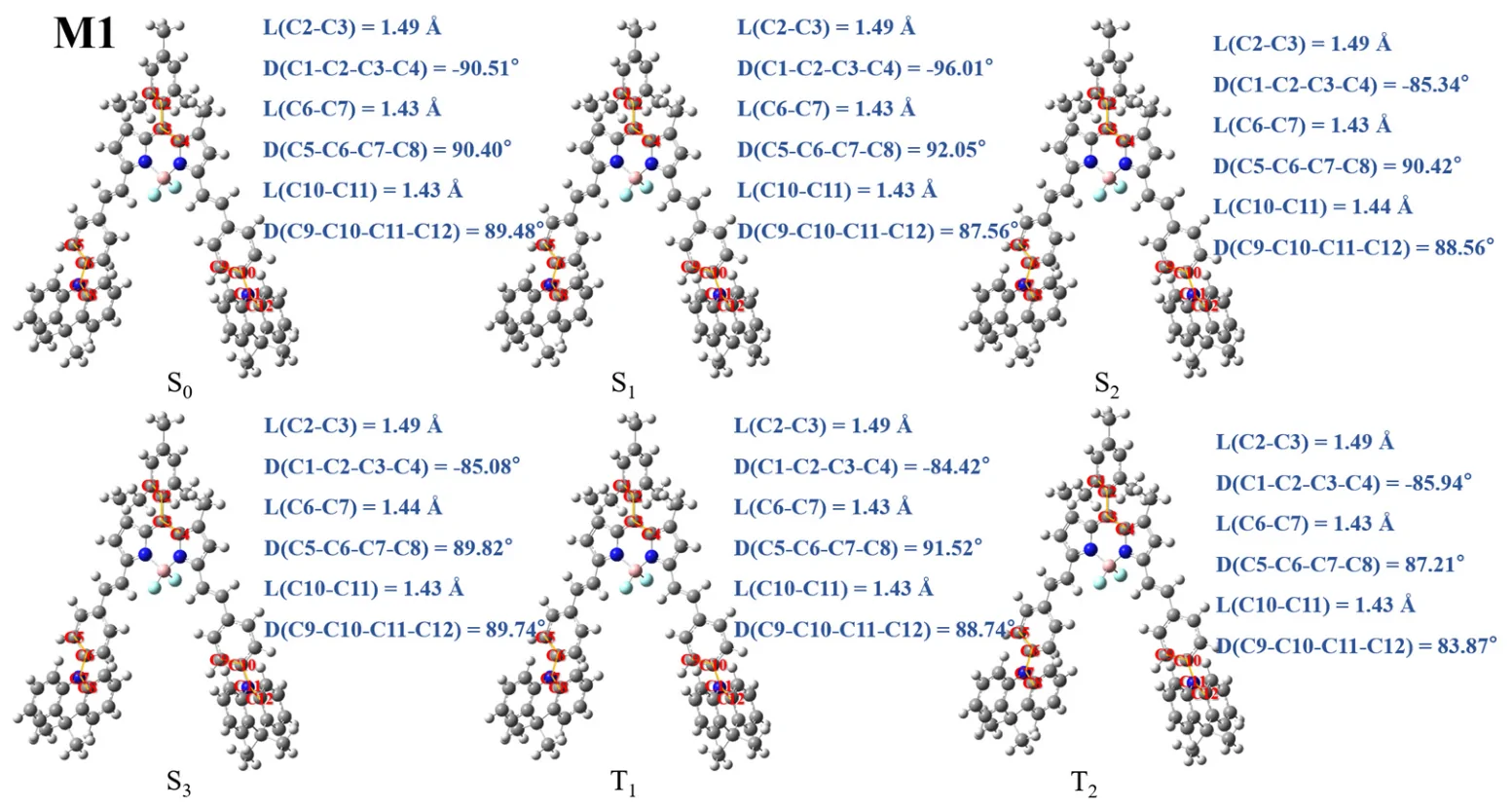
The S_0_ state, the S_n_ state and the T_n_ state geometric structures of M1.

**Figure 3:**
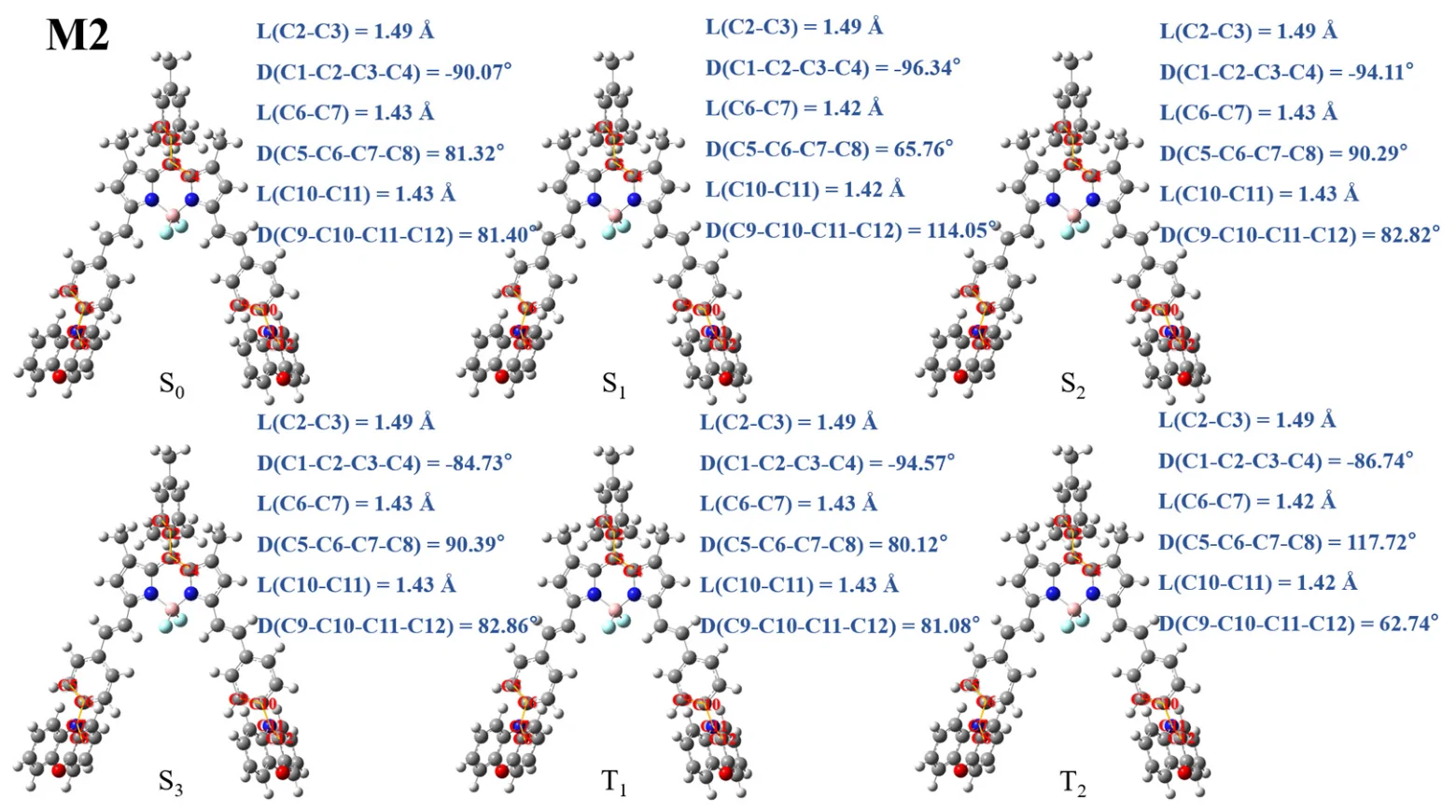
The S_0_ state, the S_n_ state and the T_n_ state geometric structures of M2.

**Figure 4:**
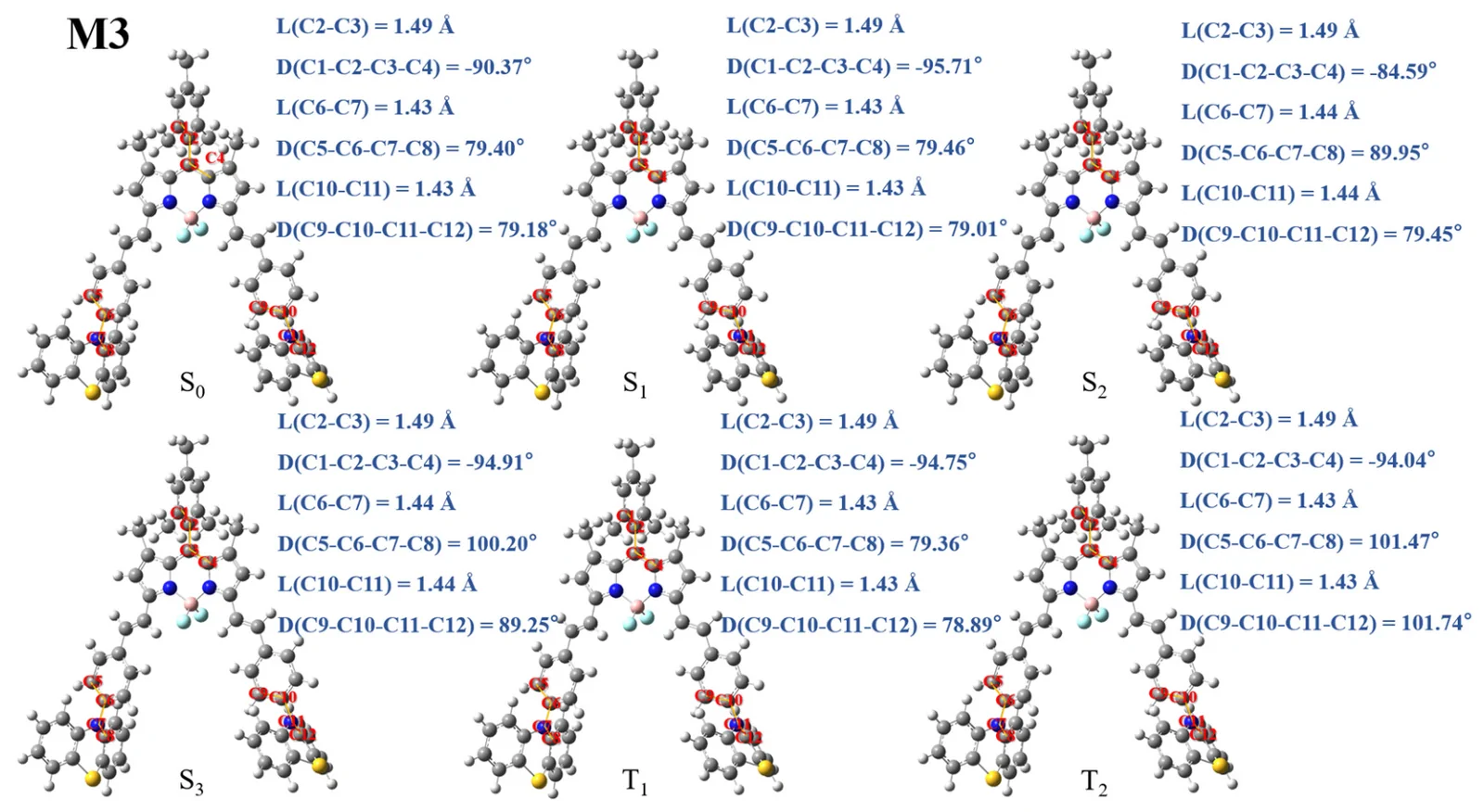
The S_0_ state, the S_n_ state and the T_n_ state geometric structures of M3.

To investigate the influence of geometric configuration on the photophysical properties of M1, M2, and M3 (Figure 1), we optimized their molecular structures and systematically analyzed key structural parameters. The optimized geometries and major bond parameters of the three molecules in the S_0_, S_n_ (singlet), and T_n_ (triplet) states are presented in Figure 2, Figure 3 and Figure 4. Owing to intramolecular steric hindrance, the dihedral angle between the distyryl-BODIPY core and the *meso*-attached phenyl ring (C1–C2–C3–C4) approaches 90° in all three molecules across different electronic states. Furthermore, we examined the evolution of the donor–acceptor dihedral angles (C5–C6–N1–C7 and C8–C9–N2–C10) on both sides of M1–M3 in the S_0_, S_1_, S_2_, S_3_ singlet states and T_1_, T_2_ triplet states. The results reveal significant molecular differences and excited-state dependence in these dihedral angles: M1 maintains both angles close to 90° in all electronic states, exhibiting a stable perpendicular conformation with strong structural rigidity, whereas M2 and M3 are more sensitive to excitation, showing noticeable twisting upon electronic transitions with varying degrees of conformational rotation among different singlet and triplet states, indicating good conformational tunability. A closer comparison shows that for M1, both dihedral angles remain near 90° in S_2_ and S_3_; for M2, only the C5–C6–N1–C7 angle approaches 90° in S_2_ and S_3_; and for M3, the C5–C6–N1–C7 angle is closer to 90° in S_2_, while the C8–C9–N2–C10 angle is closer to 90° in S_3_. Such perpendicular conformations facilitate more efficient charge transfer, effectively modulate molecular orbital angular momentum, and induce adaptive adjustments in spin angular momentum to conserve total angular momentum, thereby promoting the ISC process and providing structural support for efficient singlet–triplet transitions and subsequent singlet-oxygen generation.

To further investigate the modulating effect of donor structure on the electron-donating properties of M1–M3, we calculated the oxidation potentials of their respective donor moieties. Previous studies have established that a higher oxidation potential corresponds to a weaker electron-donating ability of the donor [45]. The data in Table 1 show that M2 exhibits the lowest oxidation potential, indicating that its phenoxazine donor possesses the strongest electron-donating performance among the three. From the perspective of donor structural features, the dimethylacridine donor in M1 contains only a single nitrogen heteroatom, whereas the donor systems of M2 and M3 incorporate two types of heteroatoms; the introduction of heteroatoms effectively enhances the electron-supplying capability of the donor units. Based on the calculated oxidation potentials, the electron-donating ability of the donors follows the order M2 > M3 > M1. Furthermore, M1–M3 all maintain near-perpendicular molecular conformations in both S_2_ and S_3_ excited states. This conformational feature, in synergy with the excellent electron-donating properties, effectively promotes charge separation and facilitates the formation of ^1^CT states, thereby providing a favorable structural and electronic foundation for the ^1^CT → ^3^LE intersystem crossing process in these systems.

### 3.2. Frontier Orbital Analysis and Fluorescence Rate Constants of M1–M3

**Figure 5:**
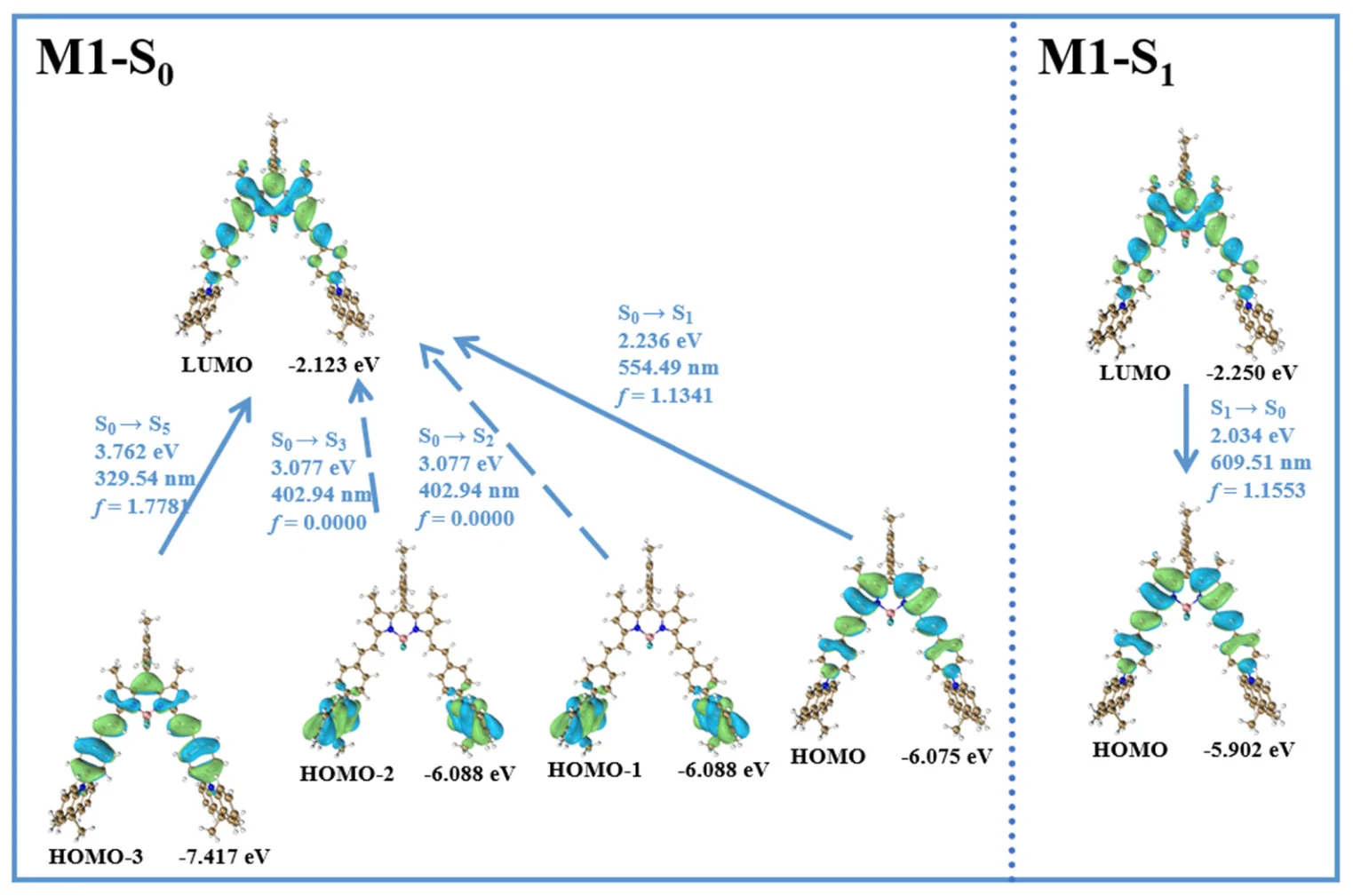
Frontier orbitals of the S_0_ state and the S_1_ state for M1.

**Figure 6:**
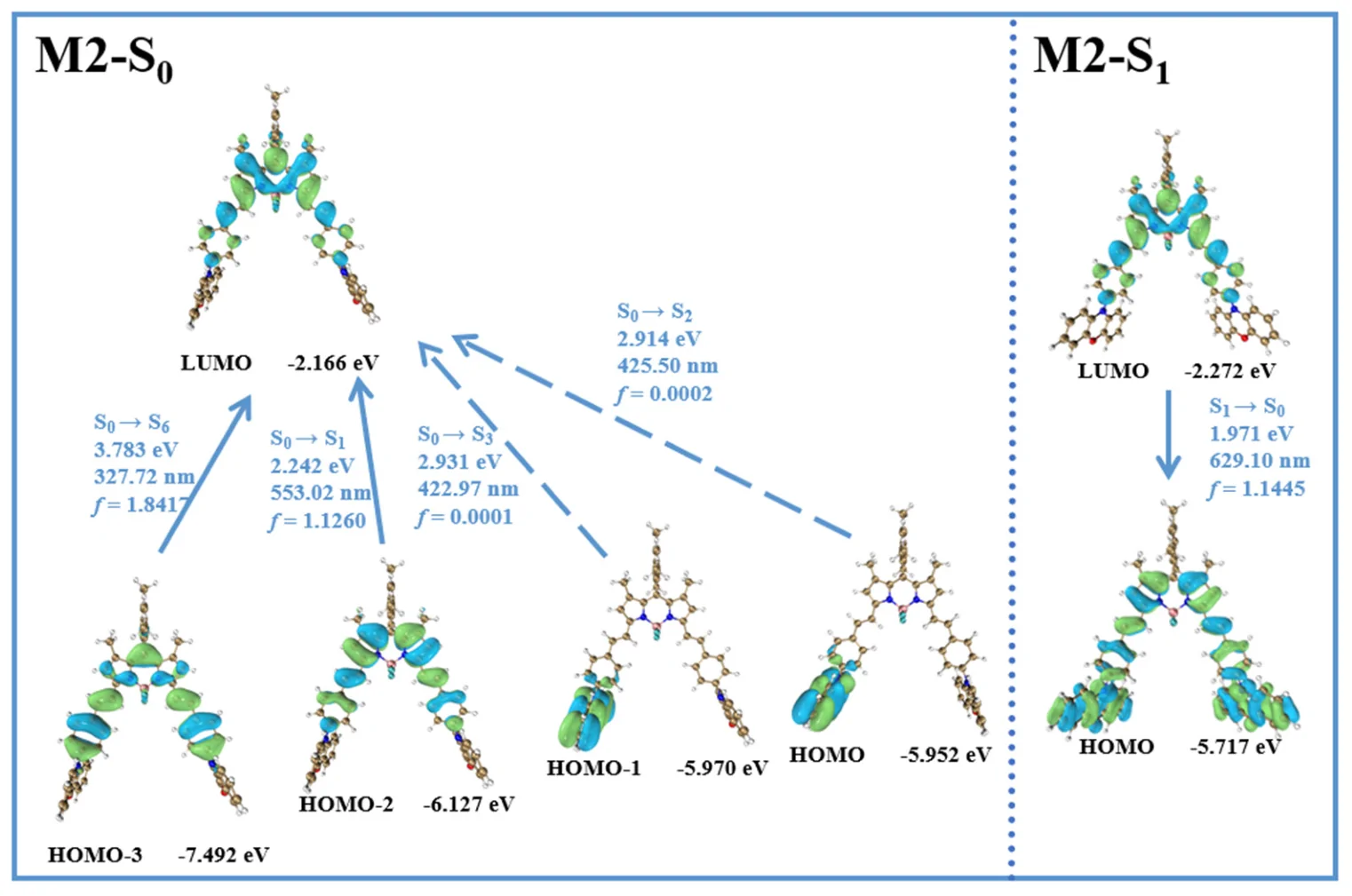
Frontier orbitals of the S_0_ state and the S_1_ state for M2.

**Figure 7:**
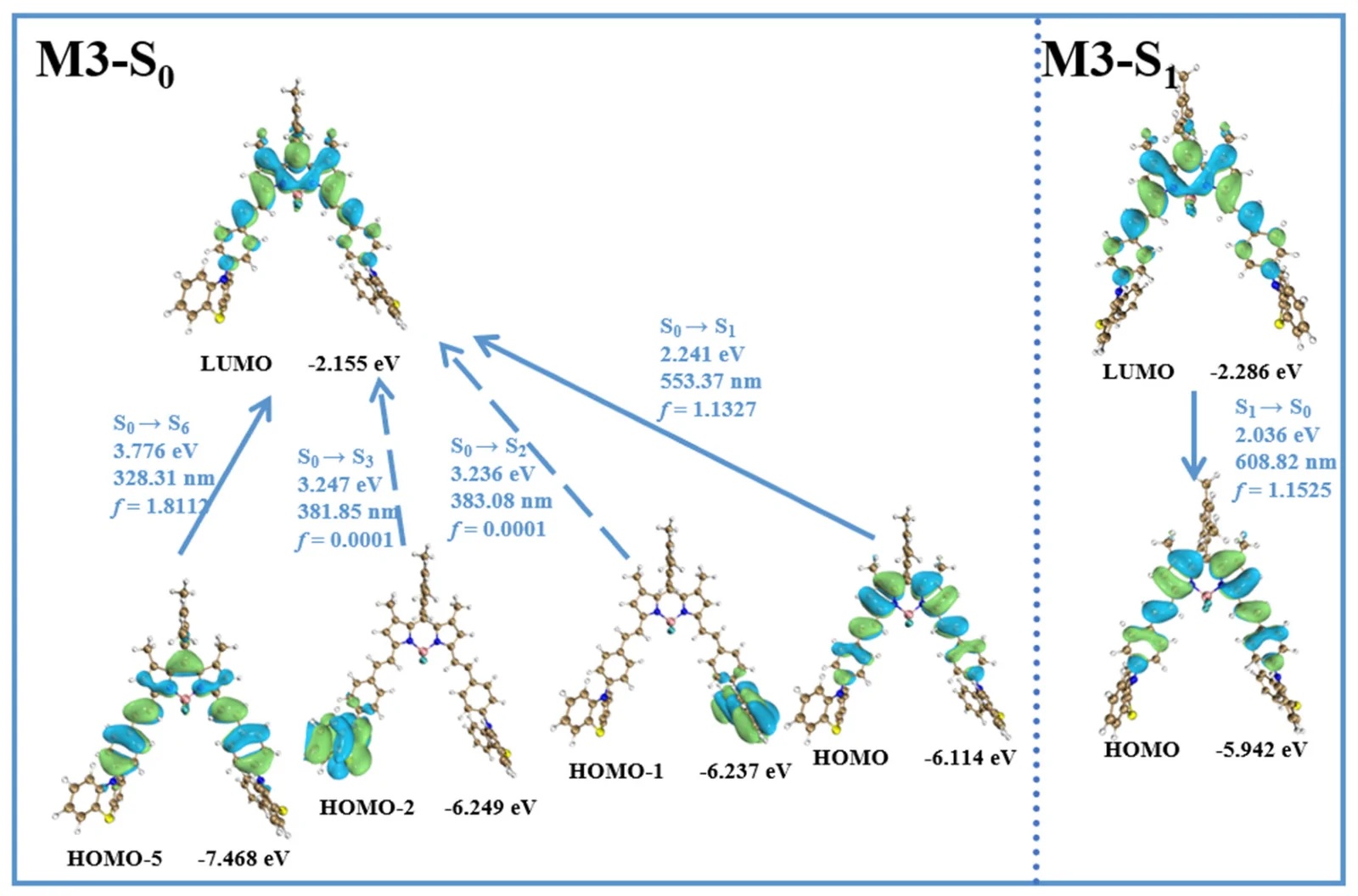
Frontier orbitals of the S_0_ state and the S_1_ state for M3.

TD-DFT calculations were performed to determine the absorption and emission wavelengths, frontier molecular orbitals, and low-lying excited states of D–A photosensitizers M1–M3 (Figure 5, Figure 6 and Figure 7). Analysis of the ground-state orbitals and excitation characteristics reveals a common pattern: high-energy excitations exhibit large oscillator strengths, whereas the S_0_ → S_2_ and S_0_ → S_3_ transitions show essentially zero oscillator strengths. This indicates that photoexcitation preferentially populates higher-lying singlet states, which then undergo internal conversion to S_2_ or S_3_ before proceeding to subsequent photophysical events. For the S_1_ state, the radiative S_1_ → S_0_ decay is dominated by the LUMO (lowest unoccupied molecular orbital) → HOMO (highest occupied molecular orbital) transition, with significant orbital overlap that ensures efficient radiative relaxation and consequently high fluorescence quantum yields—a trend fully consistent with experimental observations. In addition, the S_5_ and S_6_ states were also computed and will appear in the subsequent energy level diagram; however, they are not involved in the intersystem crossing pathways discussed below because their charge-transfer character and spin–orbit coupling are less favorable. The radiative rate constants (k_r_), calculated using Equation (1) [45] and listed in Table 2, reveal that M1 possesses the largest k_r_, in agreement with its highest fluorescence quantum yield, while M3 shows a slightly higher k_r_ than M2, mirroring the order of their experimental fluorescence performances. Collectively, these results demonstrate that variations in donor structure primarily affect the radiative efficiency, yet all three molecules share the same fundamental excitation–deactivation pathway.

(1) $$k_{r}=fE^{2}/1.499$$

where *kr* represents the radiative decay rate constant of the singlet excited state, with units of s^−1^; *f* is the oscillator strength corresponding to the S_0_ → S_1_ vertical transition, dimensionless; *E* denotes the vertical excitation energy of the first singlet excited state, in eV. The constant 1.499 is a fixed conversion factor built in the MOMAP program (version 2021B) for unit transformation from atomic units to international standard units.

To investigate whether the lone-pair electrons on heteroatoms (N, O, S) are involved in nπ ↔ ππ transitions when the molecules relax from the S_2_ and S_3_ states to the T_2_ excited state, we analyzed the excitation characters of the S_2_, S_3_, and T_2_ states for all three molecules via natural transition orbitals (NTOs), as summarized in Table 3. The results show that all these excitations are predominantly π → π* in nature; the lone-pair electrons of the heteroatoms participate only in π-conjugation with the donor moieties and do not give rise to n → π* excitation modes. Furthermore, when the isosurface value was increased to 0.04, the occupied NTOs of the S_2_, S_3_, and T_2_ states still clearly exhibit π-orbital characteristics, and no ISC promoted by nπ ↔ *ππ* transitions was observed throughout the entire analysis.

### 3.3. Electronic Excitation Characteristics of M1–M3

**Figure 8:**
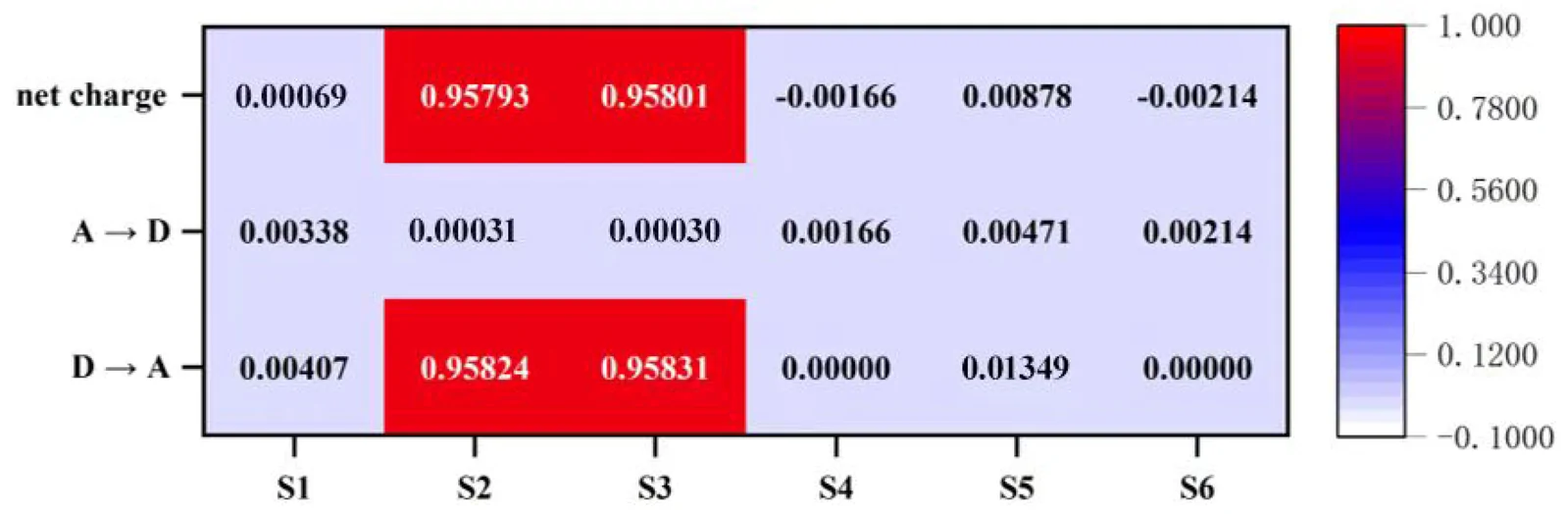
Thermal map of charge transfer (e) between donor and acceptor in M1 at S_1_–S_6_ states.

**Figure 9:**
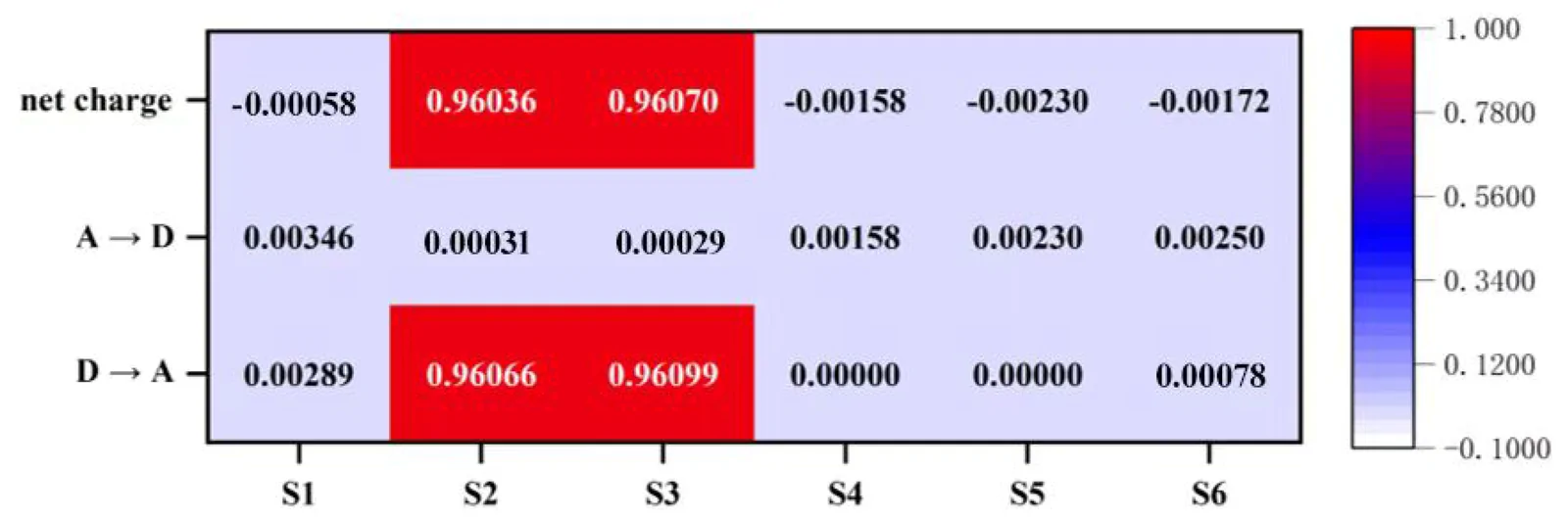
Thermal map of charge transfer (e) between donor and acceptor in M2 at S_1_–S_6_ states.

**Figure 10:**
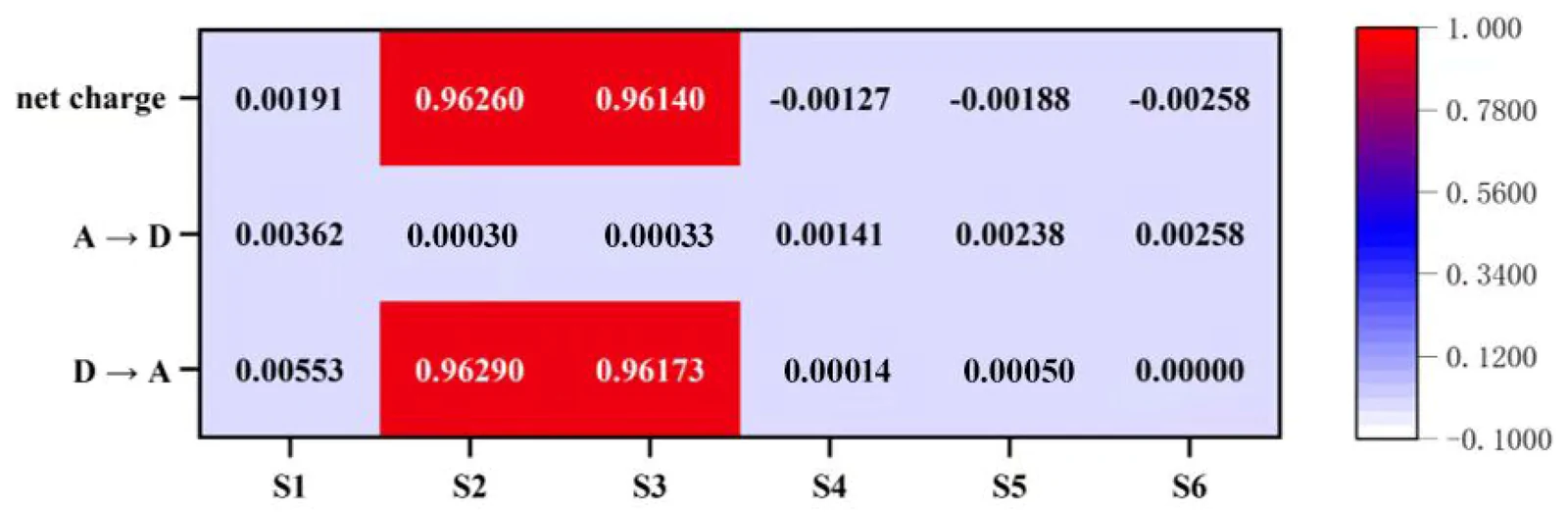
Thermal map of charge transfer (e) between donor and acceptor in M3 at S_1_–S_6_ states.

To better characterize the electronic nature of the singlet excited states in these D–A photosensitizers, we computed the net charge transfer between donor and acceptor moieties for each excited state and visualized the results as heatmaps (Figure 8, Figure 9 and Figure 10). For M1, the S_2_ and S_3_ states exhibit substantially higher net charge transfer (indicated by redder colors) than S_1_, S_4_, S_5_, and S_6_, which show negligible transfer, revealing that S_2_ and S_3_ possess strong charge-transfer character. The same trend is observed for M2 and M3: only their S_2_ and S_3_ states display significant intramolecular charge transfer, whereas all other singlet states have near-zero net charge transfer. Accordingly, the S_2_ and S_3_ states of all three molecules are unequivocally identified as charge-transfer singlet (^1^CT) states.

**Figure 11:**
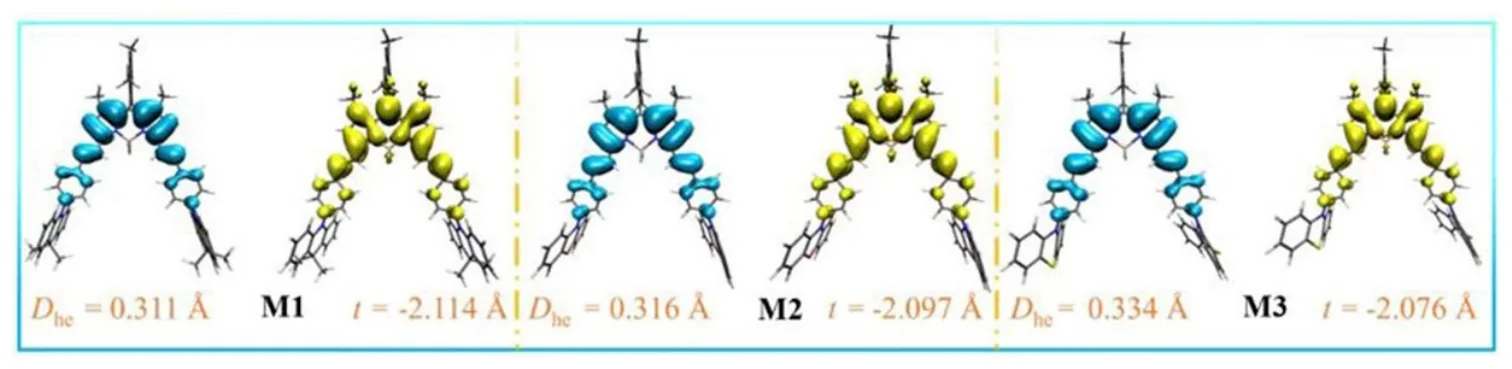
The hole (blue)–electron (yellow) diagram in S_1_ state of M1–M3.

**Figure 12:**
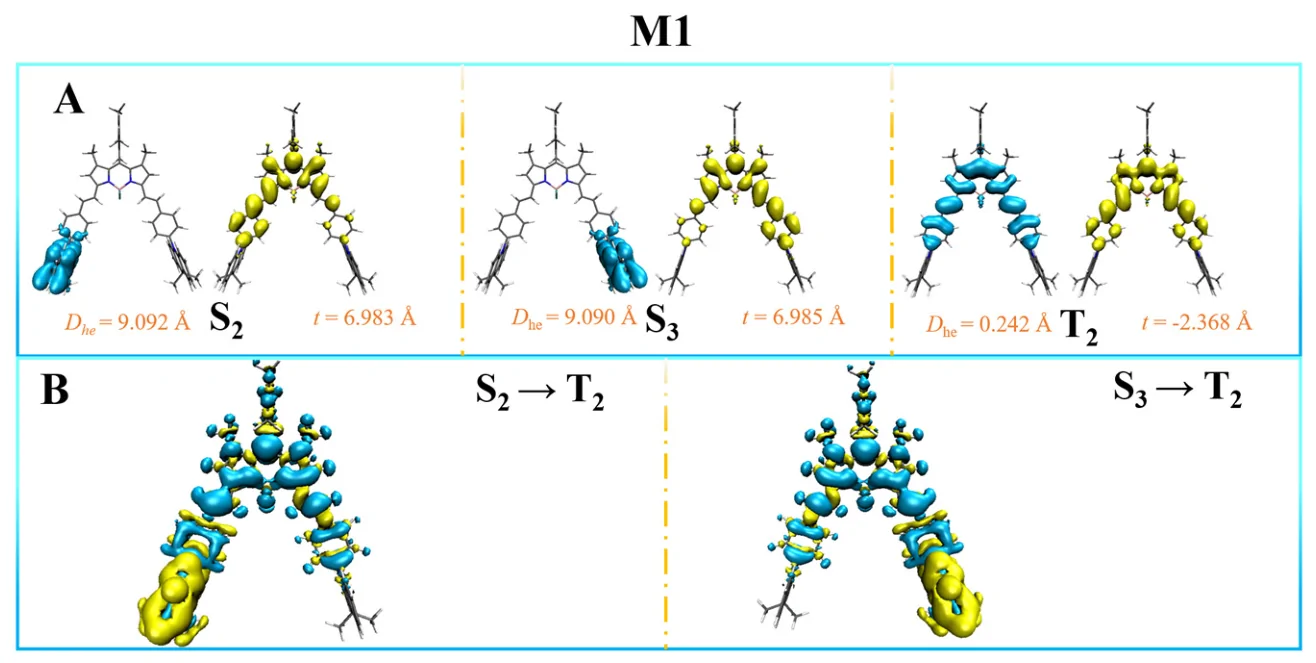
(**A**) The hole (blue)–electron (yellow) diagram of M1 and (**B**) the electron density difference between the S_2_ state, the S_3_ state and the T_2_ state diagram of M1 (blue represents a decrease in electron density, and yellow represents an increase in electron density).

**Figure 13:**
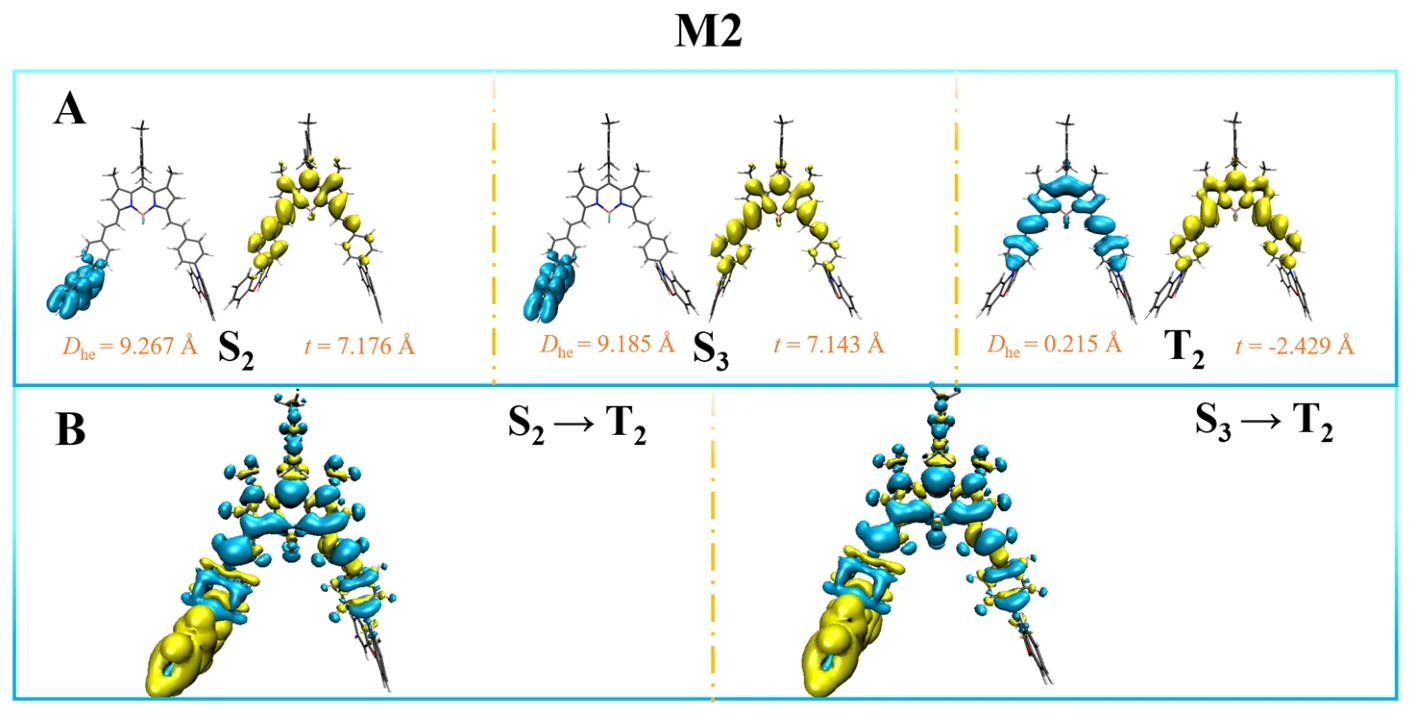
(**A**) The hole (blue)–electron (yellow) diagram of M2 and (**B**) the electron density difference between the S_2_ state, the S_3_ state and the T_2_ state diagram of M2 (blue represents a decrease in electron density, and yellow represents an increase in electron density).

**Figure 14:**
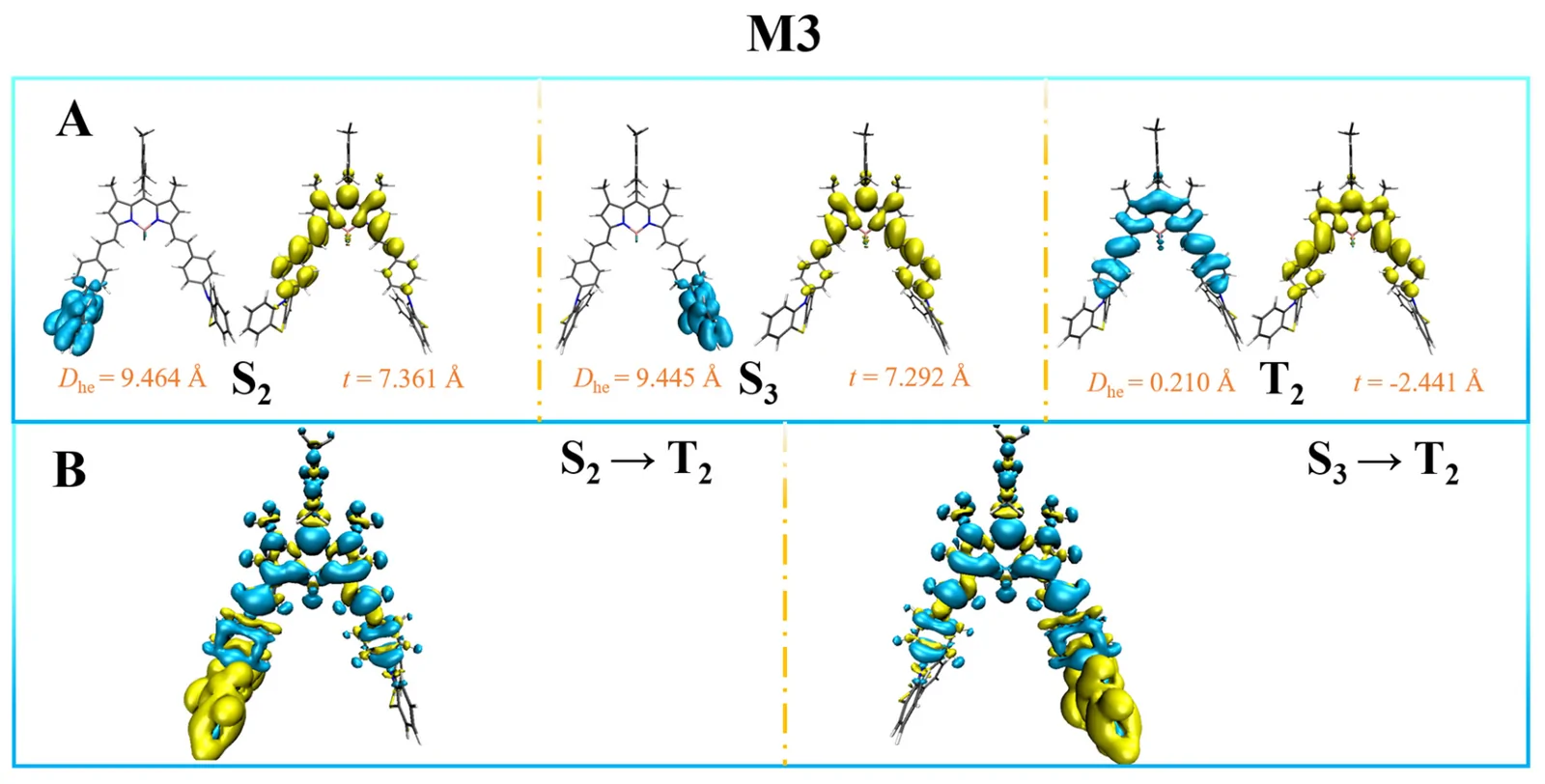
(**A**) The hole (blue)-electron (yellow) diagram of M3 and (**B**) the electron density difference between the S_2_ state, the S_3_ state and the T_2_ state diagram of M3 (blue represents a decrease in electron density, and yellow represents an increase in electron density).

To visualize the relationship between charge transfer and electron distribution, we analyzed the hole–electron distributions of the S_1_ state for M1–M3 (Figure 11). For all three molecules, both holes and electrons are predominantly localized on the acceptor moiety, with substantial spatial overlap and negligible centroid separation. This pattern indicates that the S_0_ → S_1_ excitation is locally excited (LE) in nature, confirming the assignment of S_1_ as an LE state, which is fully consistent with the near-zero net charge transfer observed for S_1_. An energy comparison reveals that the S_2_ and S_3_ states are energetically close to T_2_ for each molecule, providing a favorable basis for interstate transitions. To elucidate the charge-transfer behavior among these states, we further examined the hole–electron distributions and electron density difference maps for S_2_, S_3_, and T_2_ (Figure 12, Figure 13 and Figure 14). For the S_0_ → S_2_ transition, all three molecules exhibit large hole–electron centroid distances and pronounced charge-transfer character, with significant spatial separation and minimal orbital overlap, typical of intramolecular charge-transfer excitations, thus confirming S_2_ as a ^1^CT state. The S_0_ → S_3_ transition follows the same pattern, with equally large centroid separations and clear charge-transfer features, establishing S_3_ as also a ^1^CT state. In contrast, the T_2_ state shows much smaller centroid distances and highly overlapping hole–electron distributions with negligible charge separation, characteristic of a local excitation, indicating that T_2_ is a ^3^LE state for M1–M3. The electron density difference maps further reveal that during the S_2_ → T_2_ and S_3_ → T_2_ transitions, electron redistribution and recombination occur. Collectively, the hole–electron and electron density difference analyses demonstrate that both ISC pathways involve an evolution from ^1^CT to ^3^LE, with a charge-transfer–recombination process that effectively modulates molecular orbital angular momentum and electron spin, overcomes the spin-forbidden barrier, and thereby facilitates efficient intersystem crossing.

**Figure 15:**
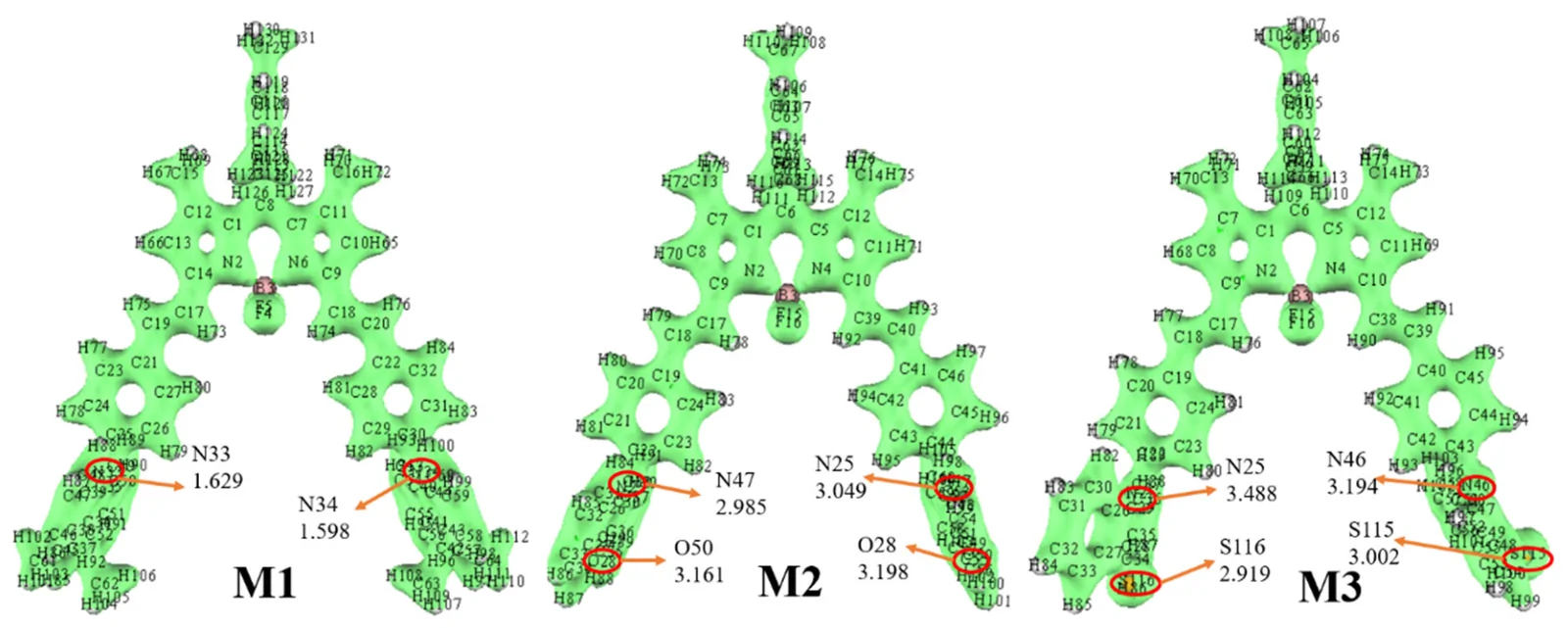
π-electron density isosurfaces (0.09) and the net π electron charge transfer (e) for M1–M3.

The π-electron characteristics of these D–A-type π-conjugated photosensitizers were analyzed. As shown in Figure 15, where green regions denote π-electron distribution, the π-electrons of all three molecules are delocalized over the entire molecular framework, indicating a highly conjugated system. The donor moieties of M1–M3 contain heteroatoms (N, O, S), and the calculated net π-electron charges on these heteroatoms are notably large, suggesting that the heteroatoms effectively participate in the π-conjugation of the donors and thus enhance their electron-donating capability.

### 3.4. Intersystem Crossing Processes of M1–M3

**Figure 16:**
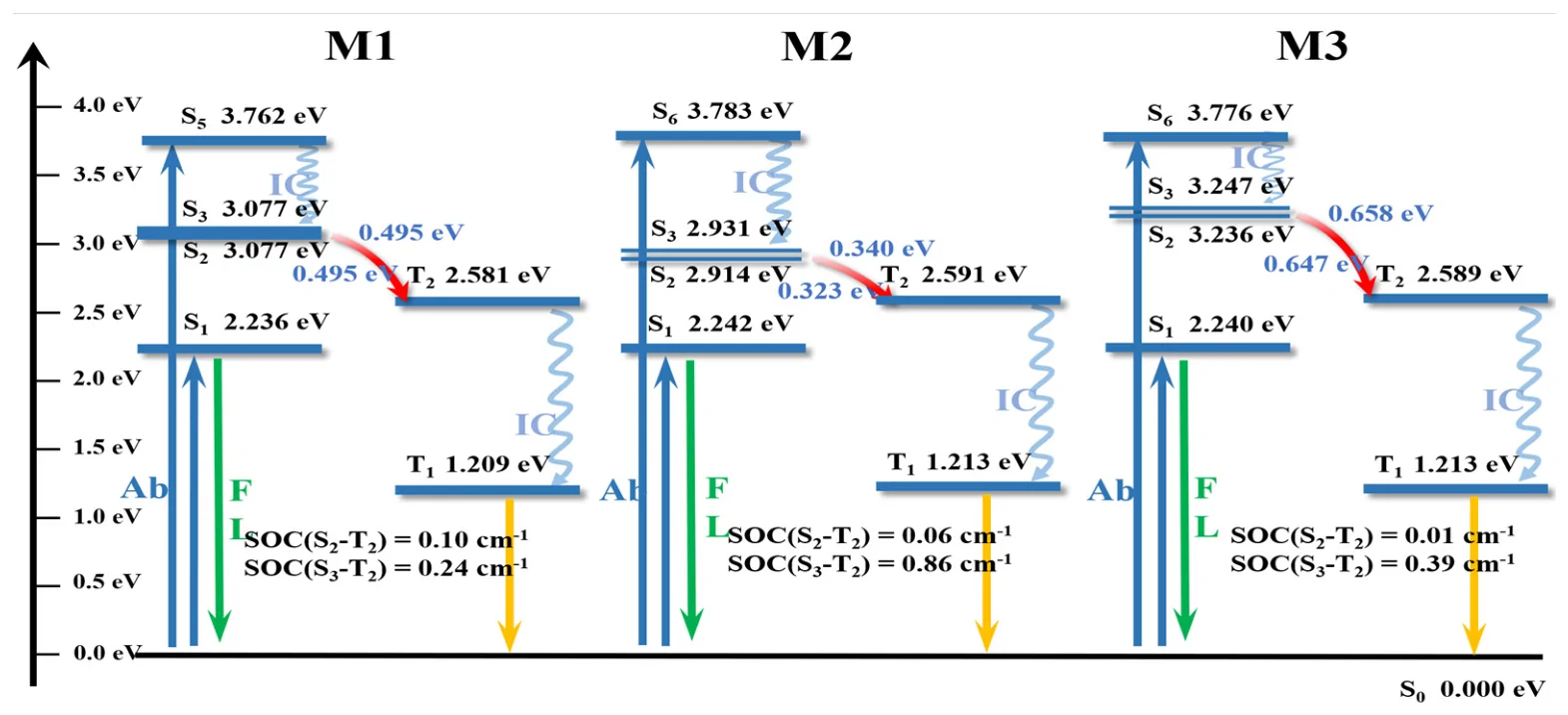
The energy levels of M1, M2 and M3 as well as the corresponding values of SOC.

It is well established that efficient triplet–triplet energy transfer from a photosensitizer to molecular oxygen requires the triplet energy of the sensitizer to exceed 0.98 eV, which corresponds to the experimentally measured energy gap between the triplet ground state (^3^O_2_) and the first singlet excited state (^1^O_2_) of oxygen [46]. Only when this thermodynamic criterion is met can the photosensitizer effectively generate reactive singlet oxygen. Upon photoexcitation, the higher-lying singlet states (e.g., S_5_ and S_6_) are initially populated due to their large oscillator strengths; these states then undergo rapid internal conversion to the lower-lying S_2_ and S_3_ states. Based on the excitation characteristics, S_2_ and S_3_ are both ^1^CT states, and ISC in M1–M3 proceeds via the ^1^CT → ^3^LE mechanism from both states. Efficient ISC requires small ΔE_ST_ and large SOC to maximize *k_isc_*; thus S_2_ → T_2_ and S_3_ → T_2_ are identified as the operative channels. Energy diagrams incorporating SOC values (Figure 16) reveal that M1 has quasi-degenerate S_2_/S_3_ states. The quasi-degeneracy arises from their very similar excitation characters and close energetic spacing, leading to strong state mixing that effectively merges the two potential ISC pathways (S_2_ → T_2_ and S_3_ → T_2_) into a single effective channel. To avoid ambiguity, we identify the dominant ISC channels using two criteria: (i) the spin–orbit coupling (SOC) exceeds 0.1 cm^−1^, and (ii) the energy gap ΔE_S−T_ is smaller than 1.0 eV. Applying these criteria, only the S_3_ → T_2_ channel in M1 satisfies both conditions, whereas M2 and M3 satisfy them for both S_2_ → T_2_ and S_3_ → T_2_. Consequently, M1 has an effective single ISC channel with ΔE_S3-T2_ = 0.495 eV, SOC = 0.24 cm^−1^, and *k_isc_* = 3.948 × 10^4^ s^−1^ (Table 4)—the lowest among the three, matching its experimentally lowest Φ_Δ_. M2 (phenoxazine) and M3 (phenothiazine) offer stronger electron donation than M1 and provide two synergistic ISC channels. M2 shows ΔE = 0.340 eV and SOC = 0.86 cm^−1^, while M3 gives ΔE = 0.658 eV and SOC = 0.39 cm^−1^; despite the sulfur atom in M3, M2’s smaller energy gap leads to superior SOC. The corresponding *k_isc_*_(*S3-T2*)_ values are 1.376 × 10^6^ s^−1^ (M2) and 1.504 × 10^5^ s^−1^ (M3), both far exceeding that of M1, with secondary S_2_ → T_2_ contributions further enhancing ISC. Consequently, M2 exhibits the strongest electron donation, highest ISC efficiency, and greatest singlet-oxygen yield, consistent with experimental Φ_Δ_ trends and confirming that strengthening donor ability is an effective strategy to boost ISC and photosensitizing performance in D–A systems.

## 4. Conclusions

In this work, we have systematically investigated the structure–property relationships in a series of distyryl-BODIPY photosensitizers (M1–M3) featuring different donors at the *para* positions of both styryl arms, with a particular focus on their dual functionality for fluorescence imaging and photodynamic therapy. Our calculations reveal that, for the three derivatives investigated here, the donor architecture critically modulates the excited-state energetics and intersystem crossing (ISC) dynamics. Among the three derivatives, M2 (phenoxazine) exhibits the lowest oxidation potential and strongest electron-donating capacity, which leads to the largest spin–orbit coupling, the smallest S_3_–T_2_ energy gap, and the highest ISC rate, thus ensuring the most efficient triplet population. The two donor groups afford multiple intersystem crossing (ISC) channels, and both electron density difference maps and hole–electron analysis confirm that ISC in M1–M3 proceeds via a ^1^CT → ^3^LE mechanism, with two feasible pathways (S_3_ → T_2_ and S_2_ → T_2_). While M2 and M3 retain both channels, M1 suffers from quasi-degeneracy of S_2_ and S_3_, which reduces its ISC pathways to one and weakens its triplet formation. For fluorescence, the S_1_ → S_0_ radiative rate is determined by the HOMO–LUMO overlap; M1 shows the largest oscillator strength and fastest radiative decay, giving the highest fluorescence quantum yield, whereas M2 and M3 still display appreciable radiative rates suitable for imaging applications. Overall, by appropriately selecting the donor group and tuning its electron-donating strength, we effectively balance ISC efficiency and fluorescence performance within this series of distyryl-BODIPY derivatives. However, this trend should not be overgeneralized to other molecular systems, because other factors—such as energy gaps, spin–orbit coupling, molecular geometry, and vibronic coupling—may also play significant roles in determining the overall ISC efficiency. This work not only rationalizes the experimental observations but also provides a robust theoretical framework and mechanistic guidance for the molecular design of BODIPY-based photosensitizers. Since the present study is entirely computational, the conclusions should be viewed as theoretical insights and design guidelines; direct claims regarding photodynamic therapy or theranostic applications await further experimental verification.

## Figures and Tables

**Table 1:** Oxidation potential (V) for donors of M1, M2 and M3 (toluene).

	M1	M2	M3
E_OX_ (V)	2.097	1.561	1.741

**Table 2:** Radiative excitation rate constants (*k_r_*) of M1, M2 and M3.

	M1	M2	M3
*k_r_* (s^−1^)	2.075 × 10^8^	1.929 × 10^8^	2.074 × 10^8^

**Table 3:** Excitation types of the S_2_, S_3_ and T_2_ states of M1, M2 and M3.

Compound	NTO Pairs for Corresponding Transitions	Occupied NTO Orbital (Iso:0.02)	Occupied NTO Orbital (Iso:0.04)	Unoccupied NTO Orbital (Iso:0.02)	Excitation Type (*: antibonding orbital.)
M1-S_2_	NTO253 → NTO254 (99.8%)	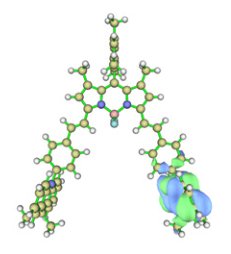	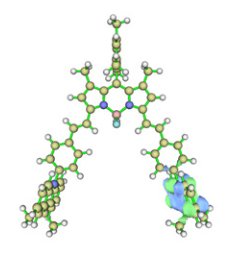	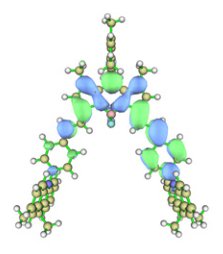	^1^(π,π*)
M1-S_3_	NTO253 → NTO254 (99.7%)	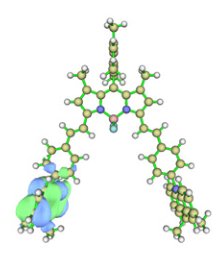	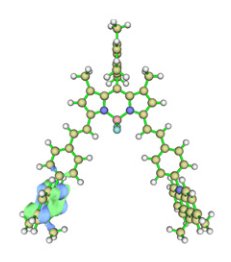	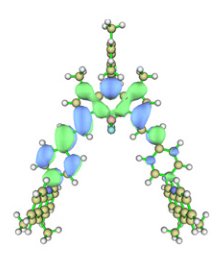	^1^(π,π*)
M1-T_2_	NTO253 → NTO254 (62.1%)	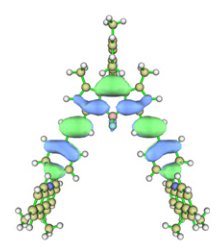	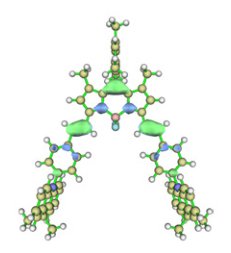	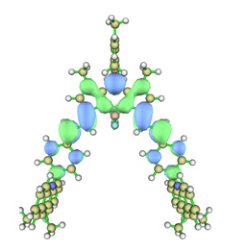	^3^(π,π*)
M2-S_2_	NTO237 → NTO238 (99.8%)	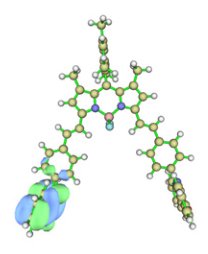	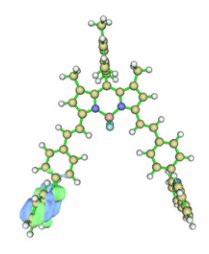	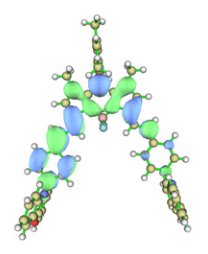	^1^(π,π*)
M2-S_3_	NTO237 → NTO238 (99.7%)	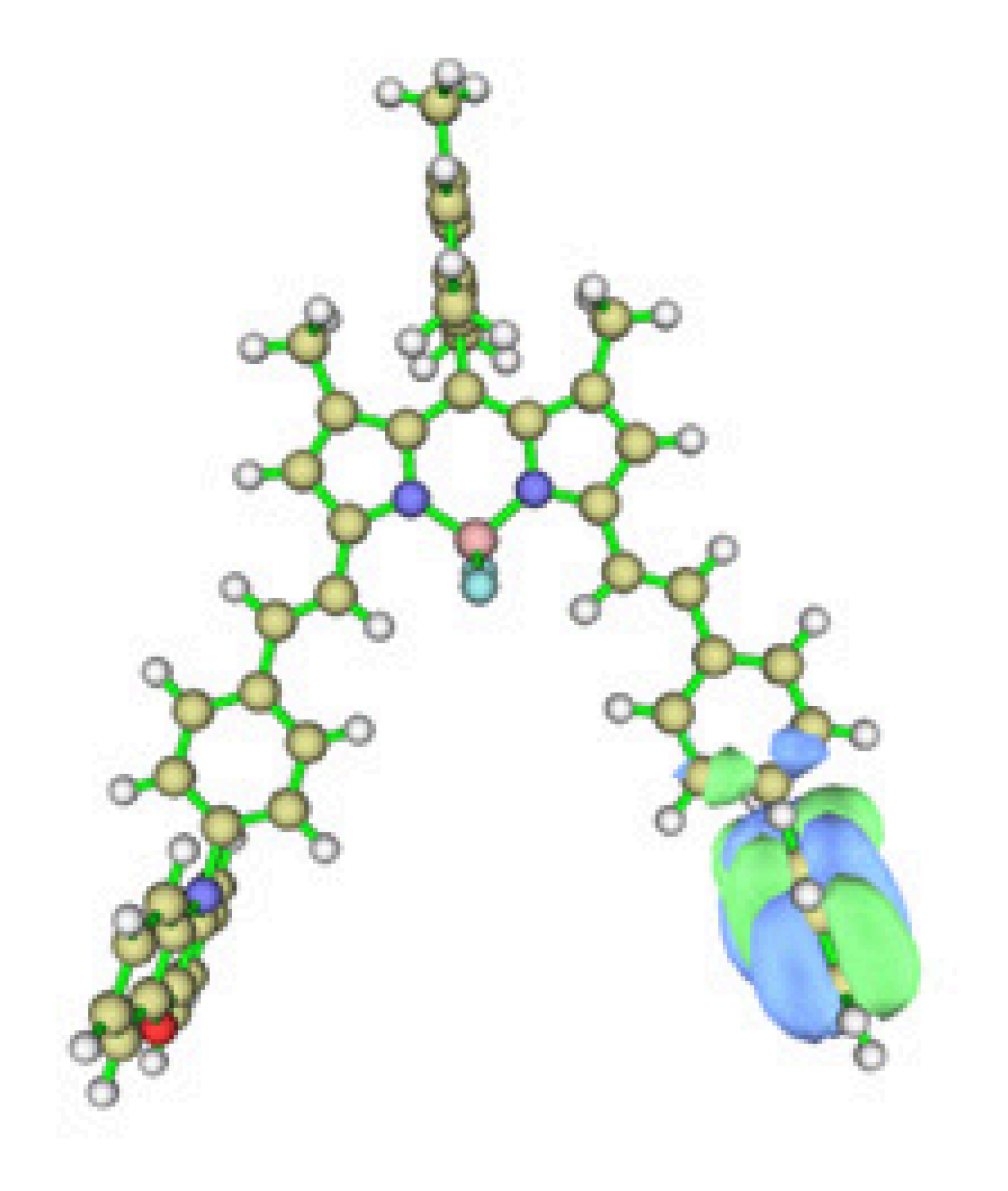	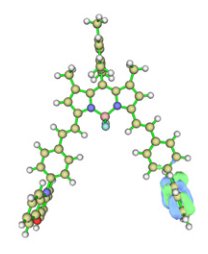	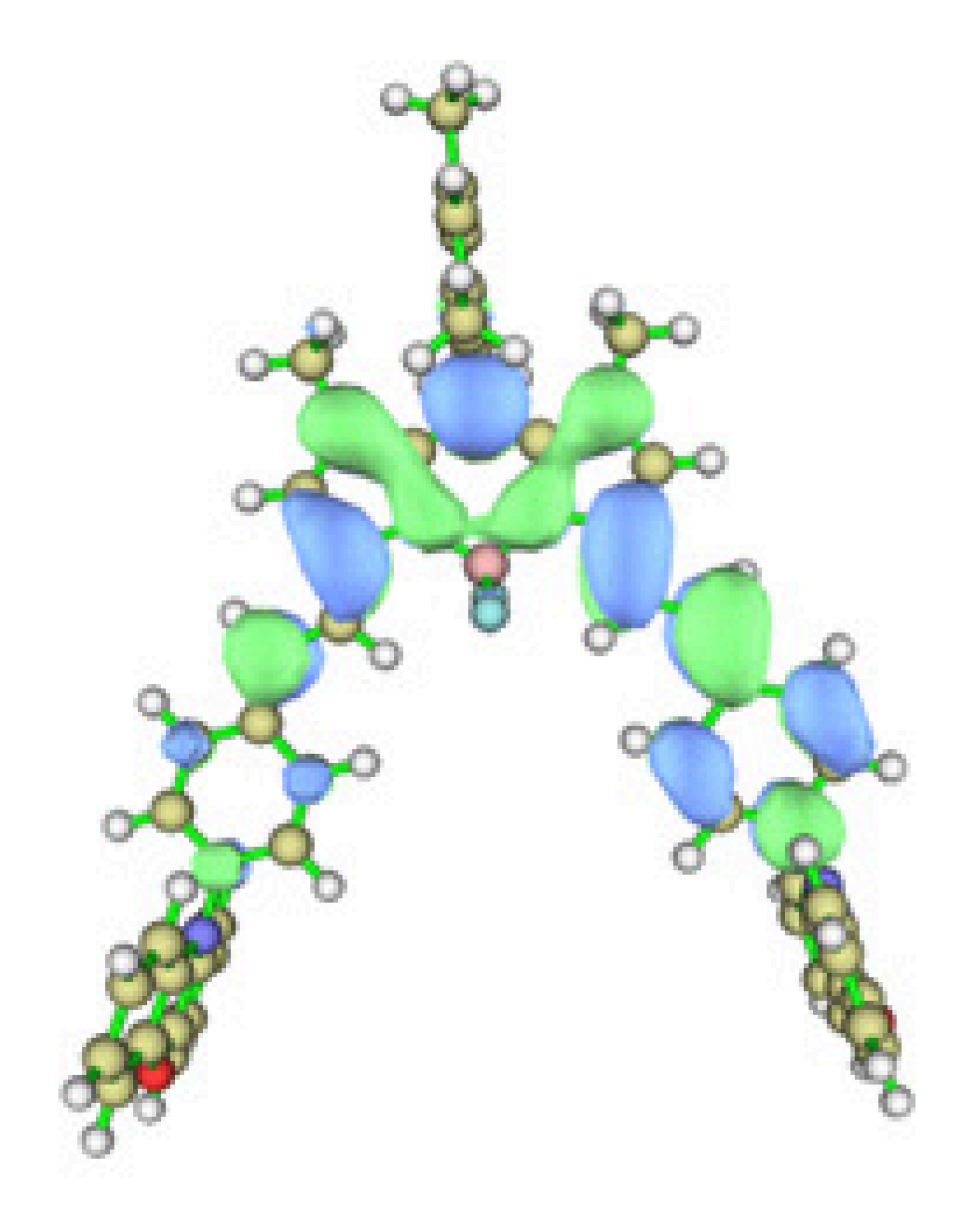	^1^(π,π*)
M2-T_2_	NTO237 → NTO238 (61.6%)	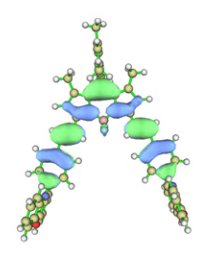	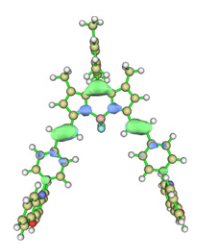	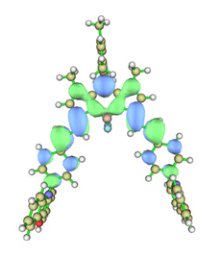	^3^(π,π*)
M3-S_2_	NTO245 → NTO246 (99.6%)	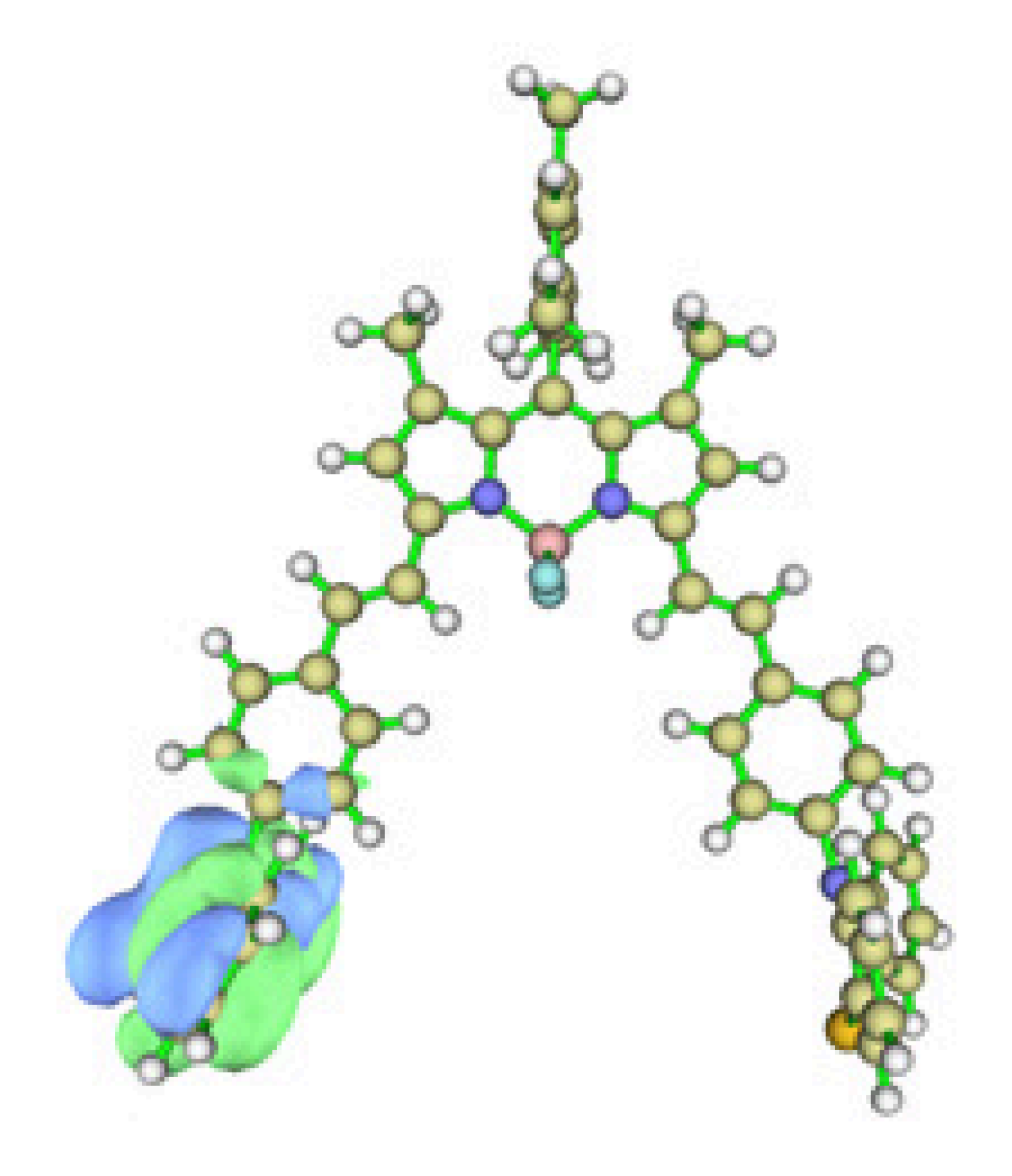	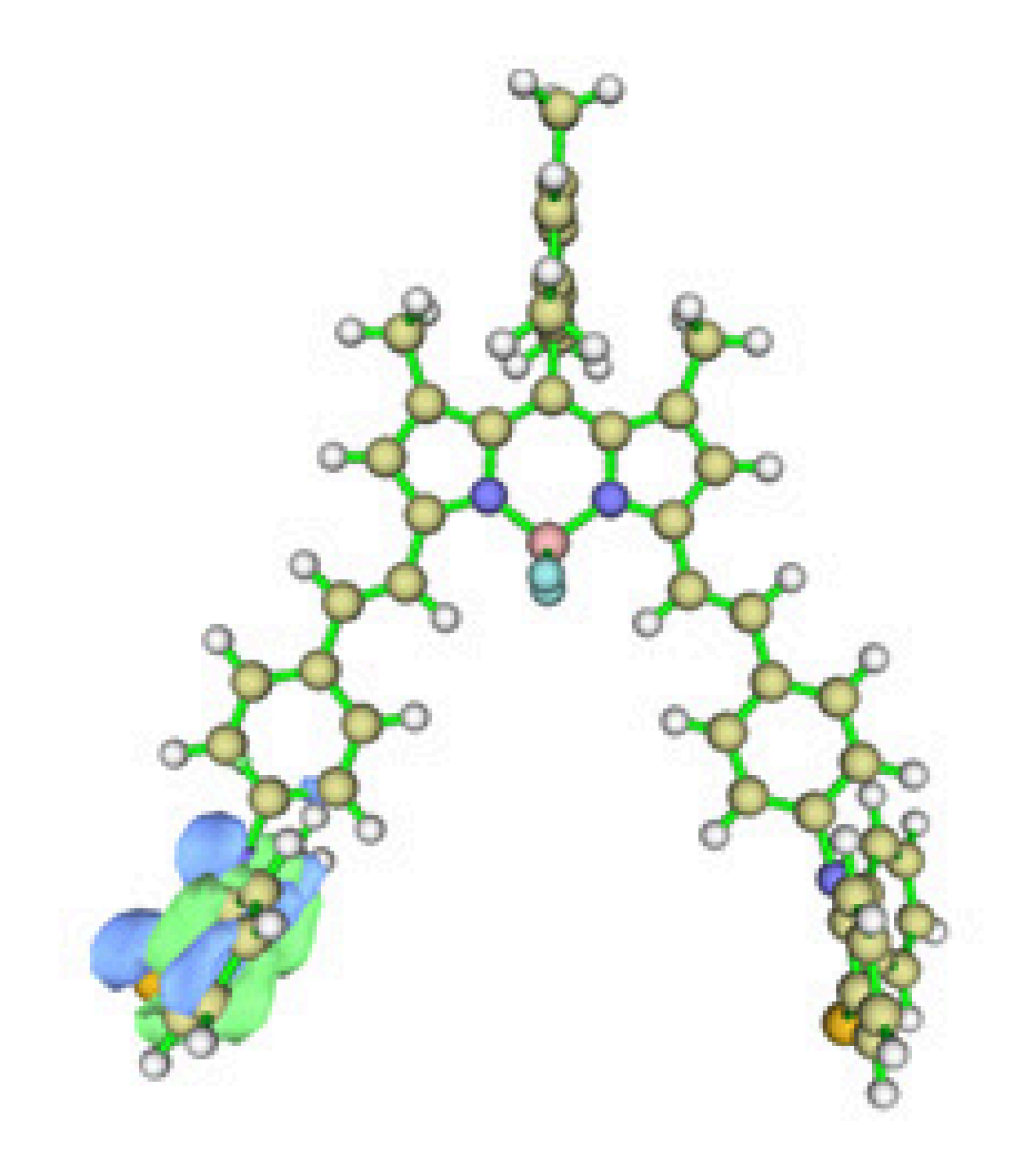	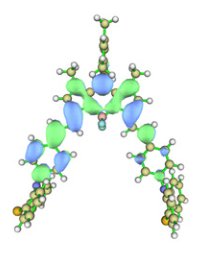	^1^(π,π*)
M3-S_3_	NTO245 → NTO246 (99.6%)	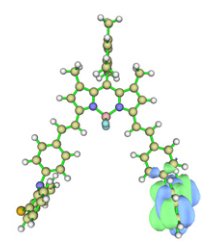	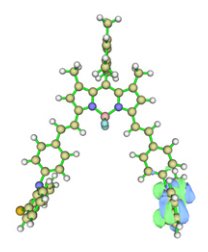	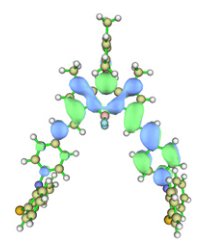	^1^(π,π*)
M3-T_2_	NTO245 → NTO246 (61.7%)	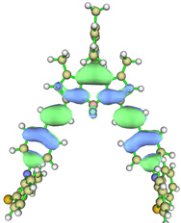	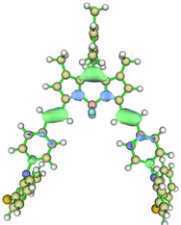	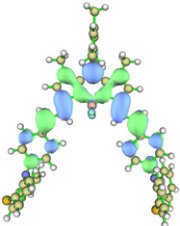	^3^(π,π*)

**Table 4:** Intersystem crossing rate constants *k_isc_* (s^−1^) for M1, M2 and M3.

	M1	M2	M3
*k_isc_*_(*S3-T2*)_	3.948 × 10^4^	1.376 × 10^6^	1.504 × 10^5^
*k_isc_*_(*S2-T2*)_	/	2.281 × 10^4^	9.888 × 10^1^

## Data Availability

The original contributions presented in this study are included in the article/Supplementary Materials. Further inquiries can be directed to the corresponding author.

## Supplementary Materials

The following supporting information can be downloaded at: https://www.mdpi.com/article/10.3390/ma19173700/s1, Table S1: Comparison of calculated and experimental photophysical parameters of M1, M2 and M3.
