# Supplementary material for: Mechanistic Study on Dual-Donor Modified Distyryl-BODIPY Photosensitizers
Source: Materials (Basel). 2026 Aug 31;19(17):3700. doi: 10.3390/ma19173700 (PMC13567294; doi:10.3390/ma19173700)
Supplement: Supplementary file 1 [file materials-19-03700-s001.zip › materials-4486600-supplementary.pdf]

Compound	$\lambda_{abs-cal}$ / nm	$\lambda_{abs-exp}$ / nm	λ_{em-cal} / nm	λ_{em-exp} / nm	$\Phi_{fluo(calc)}$	$\Phi_{fluo(exp)}$	$\Phi_{\Delta(calc)}$	$\Phi_{\Delta(exp)}$
M1	636	636	649	663	0.69	0.53	0.07	0.10
M2	638	638	663	680	0.47	0.47	0.47	0.47
M3	636	636	680	653	0.53	0.38	0.33	0.33

Table S1. Comparison of calculated and experimental photophysical parameters of M1, M2 and M3

Note: All experimental λabs, λem, Φfluo and ΦΔ values are taken from Ref. [22]; all computational results are obtained at the M06-2X/6-31G(d,p) level combined with the SMD solvation model in toluene.
